# *Ziziphora taurica* subsp. *taurica*: Analytical Characterization and Biological Activities

**DOI:** 10.3390/biom9080367

**Published:** 2019-08-14

**Authors:** Michał Tomczyk, Olcay Ceylan, Marcello Locatelli, Angela Tartaglia, Vincenzo Ferrone, Cengiz Sarikurkcu

**Affiliations:** 1 Department of Pharmacognosy, Faculty of Pharmacy, Medical University of Białystok, Białystok 15-230, Poland; 2 Department of Biology; Science Faculty, University of Mugla Sitki Kocman, Mugla 48260, Turkey; 3 Department of Pharmacy, University of Chieti–Pescara “G. d’Annunzio”, Chieti 66100, Italy; 4 Department of Analytical Chemistry, Faculty of Pharmacy, Afyonkarahisar University of Health Sciences, Afyonkarahisar 03100, Turkey

**Keywords:** biological activities, *Ziziphora taurica* subsp. *taurica*, total phenolic content, total flavonoid content, apigenin, LC–ESI–MS/MS

## Abstract

The Lamiaceae family comprises many flowering plants classified into about 236 genera. The genus *Ziziphora* is one of the well-known genera of this family and its species are important in different fields of pharmaceutical, chemical, traditional, and folk medicines. The phytochemicals present in *Ziziphora* include monoterpenic essential oils, triterpenes, and phenolic substances. The aim of this paper was to study the phytochemical profile of *Ziziphora taurica* subsp. *taurica* and compare and evaluate the biological activities of its ethyl acetate (ZTT-EtOAc), methanolic (ZTT-MeOH), and aqueous (ZTT-W) extracts based on their enzyme inhibition and antioxidant capacities. Determination of total phenolic (TPC) and total flavonoid (TFC) contents as well as biological activities were determined using spectrophotometric procedures. Subsequently, the individual phenolic compounds were detected by liquid chromatography–electrospray ionization–tandem mass spectrometry (LC–ESI–MS/MS). In total, twenty-two different phenolic compounds were identified, including apigenin, ferulic acid, and luteolin which were the most common. ZTT-MeOH extract showed the best antioxidant activity, whereas ZTT-EtOAc extract was the most effective against tyrosinase and α-amylase. *Ziziphora taurica* subsp. *taurica* represents a potential source of natural compounds with positive effects on human health.

## 1. Introduction

In recent years, the study of natural products for the discovery of new active compounds with beneficial properties for human health is growing worldwide [1]. Plants represent sources of essential oils or secondary metabolites with a wide range of therapeutic action, and using them avoids the side effects typical of synthetic drugs. Consequently, their use has become widespread, above all else, for the sake of human health [2].

Lamiaceae is one of the most important herbal families, well known for their medicinal effects [3]. The prototypical of the Lamiaceae family is represented by the *Ziziphora* species, and its world population is represented by more than 30 different species inhabiting regions of Asia, Europe, and Africa. However, their largest aggregations are in China and Kazakhstan and in some regions of Afghanistan, Anatolia, Armenia, Caucasia, Iraq, Pakistan, Syria, Turkey, Turkmenistan, and West Siberia. *Ziziphora* includes annual, perennial, herbaceous, or sub-shrub plants. The leaves are short, petiolate, or sub-sessile. The flowering period is from June to September, depending on environmental conditions [4].

The history of using plants of the genus *Ziziphora* began in ancient times. The active ingredients of these plants were usually administered as infusions against infections, hemorrhoids, hypertension, and gastrointestinal problems [5]. The first ethnopharmacological reports relate to the use of *Ziziphora* extracts as a potential medicine for wound healing and edema treatment, and as a potential antipyretic drug [6]. Many of the described species of the genus *Ziziphora*, in particular *Z. clinopodioides* L. and *Z. tenuior* L., were repeatedly prescribed in folk medicine in many countries for the treatment of colds, bronchitis, coughs, headache, diarrhea, nausea, typhus, and even cardiovascular disorders. Some of these species have been recognized not only as having effective anti-inflammatory, tranquilizing, or analgesic properties, but also as aphrodisiacs and flavorings [5].

Previously conducted studies on the phytochemical profile of species belonging to the genus *Ziziphora* have been mainly focused on the composition of its essential oils. Apart from essential oils, these species are also a rich source of other secondary metabolites, including flavonoids, derivatives of caffeic acid, fatty acids, triterpenes, and sterols [4,7], more than forty natural compounds have been isolated from different *Ziziphora* species. Among these secondary metabolites, phenolic compounds are certainly the most important due to their positive impact on health, reducing the risk of cardiovascular disease, neurodegenerative disorders, and cancer [8]. One of the best strategies to verify the activity of these compounds is to study their inhibitory capacity against various enzymes such as tyrosinase or α-amylase. Tyrosinase is a key enzyme in melanin biosynthesis that catalyzes two important reactions: the hydroxylation of L-tyrosine to 3,4-dihydroxy-L-phenylalanine (L-DOPA) and the oxidation of L-DOPA to dopaquinone followed by further conversion to melanin. The tyrosinase inhibitor plays an important role in many diseases such as skin cancer or other disorders concerning the hyperpigmentation of melanin [9]. α-Amylase is a hydrolytic enzyme that intervenes in the digestion of carbohydrates. Inhibition of this enzyme in diabetic patients leads to a decrease in plasma glucose levels [10].

**Figure 1:**
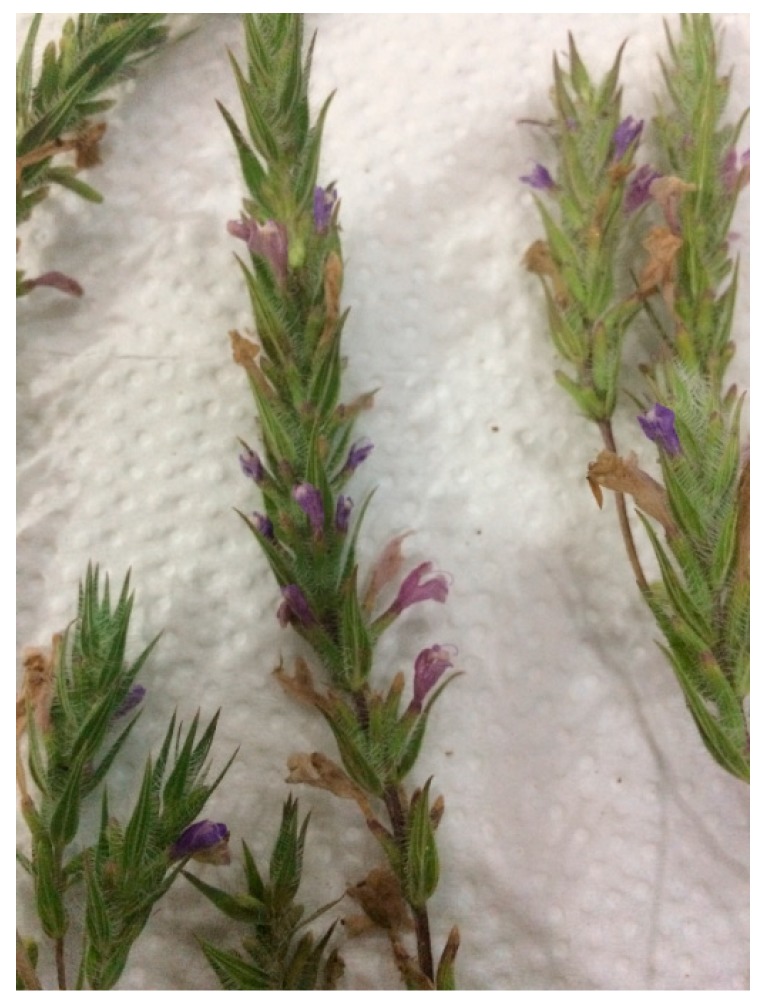
Image of the *Z. taurica* subsp. *taurica.*

Following our research on endemic plants used in traditional and folk medicine which could represent a valid source of bioactive components and their respective biological activities [11,12], the purpose of this research is to study the phytochemical profile of *Z. taurica* subsp. *taurica,* as seen in Figure 1, which has not yet been studied, and to assess its biological activity based on evaluations of its antioxidant and enzymatic activities.

## 2. Materials and Methods

### 2.1. Plant Material

The aerial parts of *Ziziphora taurica* subsp. *taurica* (Lamiaceae) were harvested from Nebiler village, Kavaklidere, Mugla-Turkey on 17 June 2018 (1074 m, 37° 27′ 10.7” N 28° 25′ 29.0” E). The species (O.1755) was identified by Dr. Olcay Ceylan and deposited at the Herbarium of University of Mugla Sitki Kocman (Mugla, Turkey). The aerial parts were air-dried in the shade for a few weeks and then cut into small pieces with a laboratory mill before further sample treatments, solvent extractions, and analyses.

### 2.2. Solvent Extraction

Ethyl acetate (ZTT-EtOAc) and methanol (ZTT-MeOH) extracts from *Z. taurica* subsp. *taurica* aerial parts were separately prepared by maceration for 24 h and then concentrated under reduced pressure. The water extract (ZTT-W) was obtained by infusion in boiling deionized water for 15 min, and the obtained solution was lyophilized [10]. Five grams of the roots were mixed with 100 mL of solvent (1:20), and agitation was set to 150 rpm. The obtained extracts were stored at +4 °C for further analysis. Extraction yields are given in Table 1.

### 2.3. Chemicals

Gallic acid, (+)-catechin, pyrocatechol, chlorogenic acid, 2,5-dihydroxybenzoic acid, 4-hydroxybenzoic acid, (−)-epicatechin, caffeic acid, syringic acid, vanillin, taxifolin, sinapic acid, p-coumaric acid, ferulic acid, rosmarinic acid, 2-hydroxycinnamic acid, pinoresinol, quercetin, luteolin, and apigenin were purchased from Sigma-Aldrich (St. Louis, MO, USA). Vanillic acid, 3-hydroxybenzoic acid, 3,4-dihydroxyphenylacetic acid, apigenin 7-glucoside, luteolin 7-glucoside, hesperidin, eriodictyol, and kaempferol were obtained from Fluka (St. Louis, MO, USA). Verbascoside, protocatechuic acid, and hyperoside were purchased from HWI Analytik (Ruelzheim, Germany).

### 2.4. Quantification of Phenolic Compounds in the Extracts

Total phenolic (TPC) and flavonoid (TFC) contents in the extracts were firstly determined spectrophotometrically as gallic acid and quercetin equivalents, respectively [13]. Phenolic composition in the extracts was then detected by liquid chromatography–electrospray tandem mass spectrometry (LC–ESI–MS/MS). Analysis was carried out by the Agilent Technologies 1260 Infinity liquid chromatography system hyphenated to a 6420 Triple Quad mass spectrometer. A Poroshell 120 EC-C18 (100 *×* 4.6 mm I.D., 2.7 µm) column was used. The mobile phases were 0.1%, *v/v* formic acid solution (Solvent A) and methanol (Solvent B), in gradient elution. Particularly, the gradient elution profile was: 0.00 min 2% B, 3.00 min 2% B, 6.00 min 25% B, 10.00 min 50% B, 14.00 min 95% B, 17.00 min 95% B, and 17.50 min 2% B. The total run time was 18 min. The column temperature was set at 25 °C. The flow rate was 0.4 mL min^−1^, and the injection volume was 2.0 μL [14,15]. Furthermore, the tandem MS conditions were an ESI source operated in negative and positive multiple reaction monitoring (MRM) mode, a capillary voltage of −3.5 kV, a gas temperature of 300 °C, a gas flow of 11 L min^−1^, and a nebulizer pressure of 40 psi. For the quali-quantitative analyses, the single compound was identified by means of retention time, MS, and MS/MS spectra with respect to the standard solution.

### 2.5. Biological Activity

Biological activities of the extracts were determined by firstly identifying their antioxidant capacity though cupric ion (CUPRAC) and ferric ion (FRAP) reducing power, free radical scavenging activity of DPPH, ABTS, a phosphomolybdenum assay, and ferrous ion chelating [16,17,18,19].

For enzymatic inhibitory activities, the extracts were tested against α-amylase and tyrosinase using the experiment previously reported [13]. In section *Supplementary Materials* (**S1**) was given analytical methods applied for polyphenolic composition (TPC, TFC), antioxidant and enzyme inhibitory activities.

The sample concentration, which decreases the initial concentration by 50% for enzyme inhibition, radical scavenging, and metal chelation tests and provides 0.500 absorbance for reducing power and phosphomolybdenum assays, was defined as IC_50_. The biological activities of the extracts were compared with those of the standards, including Trolox, ethylenediaminetetraacetic acid (disodium salt) (EDTA), kojic acid, and acarbose, used as positive controls. The biological activities of the extracts were also given as mg standard equivalent/g extract.

### 2.6. Statistical Analysis

Results were illustrated as mean ± standard deviation. Statistical analysis was performed using SPSS software v22.0. Statistical significance was tested by one-way ANOVA (Tukey test). Values were considered significant when p-value was lower than 0.05.

## 3. Results and Discussion

### 3.1. Total Phenolic (TPC) and Flavonoid (TFC) Content

Due to the different polarities of phenolic compounds, a single extraction method is not available for these compounds and different extraction solvents (ethyl acetate, methanol, and water) were used in this work. The results obtained for each solvent are shown in Table 1. As reported, the maximum yield from the raw plant material after the extraction procedure was obtained in water (ca. 14%), whereas the lowest yields were observed in ethyl acetate extract (ca. 3%). In the literature, there is no information regarding the effect of different solvents on the extraction of phytochemicals from *Ziziphora*, but it has been observed in different species [20] that the yields for these compounds are greater in aqueous extracts.

The *Ziziphora* extract TPC determination was carried out through the Folin–Ciocâlteau assay. The Folin–Ciocâlteau method measures the reduction of the reactant by phenolic compounds through the formation of a complex that can be measured at 750 nm against gallic acid as a standard. In all three extracts analyzed, phenolic compounds and flavonoids were observed. The maximum TPC was registered in the ZTT-EtOAc (34.82 ± 0.79 mg GAEs/g extract), whereas the lowest concentration was present in the ZTT-W (15.10 ± 0.45 mg GAEs/g extract). The results are shown in Table 1.

Results of the total flavonoid content (TFC) of the obtained extracts are also reported in Table 1. The highest amounts were observed for the ZTT-MeOH (37.39 mg QEs/g extract) and ZTT-EtOAc (20.87 mg QEs/g extract), in contrast to ZTT-W, which contains a lower concentration (7.08 mg QEs/g extract). This observation suggests that most of the flavonoids were taken up by the methanol or the ethyl acetate. The findings show that, in all varieties tested, the ethyl acetate and methanol extracts had a higher concentration of flavonoids in comparison to the water extract.

### 3.2. LC-ESI-MS/MS Analysis

To identify the individual phenolic compounds, a liquid chromatography–electrospray-tandem mass spectrometry analysis method was utilized [14]. In total, in *Ziziphora* extracts, twenty-five phenolic compounds were identified and reported in Table 2 with the ZTT-MeOH extract containing the highest number of compounds (21 compounds). The most common compounds are apigenin (1475.99 and 1270.90 µg/g extract, respectively, in ZTT-EtOAc extract and in the ZTT-MeOH extract), ferulic acid (1478.13 and 1540.91 µg/g extract, respectively, in ZTT-EtOAc extract and in ZTT-MeOH extract), and luteolin (5347.32 and 5339.91 µg/g extract respectively in ZTT-EtOAc extract and in ZTT-MeOH extract).

**Figure 2:**
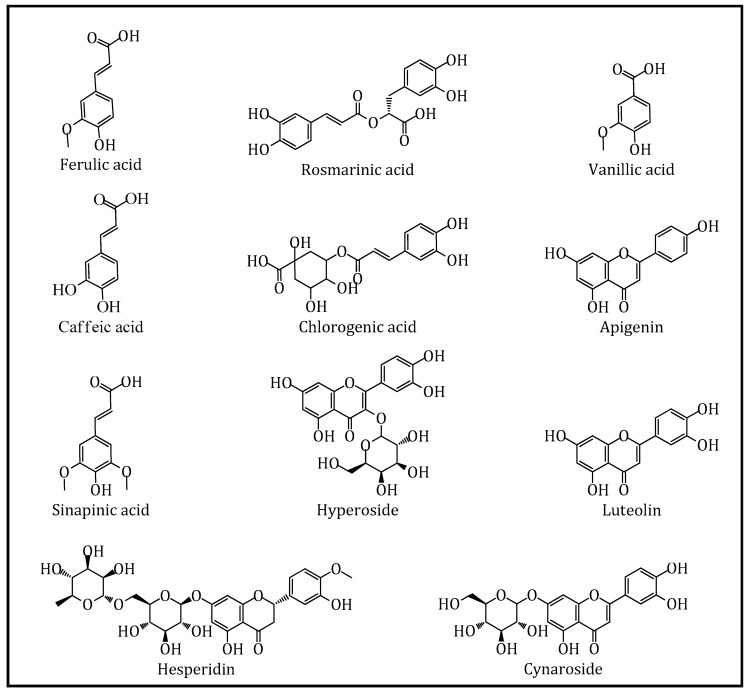
Major phenolic compounds identified in the solvent extracts from *Z. taurica* subsp. *taurica*.

The major phenolic compounds identified in *Z. taurica* subsp. *taurica* are shown in Figure 2, while the concentrations (in μg/g extract) and analytical characteristics of selected phytochemicals observed in the *Z. taurica* subsp. *taurica* extracts are reported in Table 2.

### 3.3. Biological Activities

#### 3.3.1. Antioxidant Activities

Some synthetic antioxidant compounds were found to be toxic and carcinogenic in animal models, so they need to be replaced with new and safe antioxidants of natural origin. Antioxidant activity of plant products has often been related to the phenolic content [21]. Possible mechanisms related to the antioxidant activity of these compounds include scavenging of free radicals and absorption of oxygen radicals, etc. [22].

Free radical scavenging activity of DPPH (2,2-diphenyl-1-picrylhydrazyl), ABTS (2,2’-azino-bis(3-ethylbenzothiazoline-6-sulphonic acid)) radical cation scavenging activity, the phosphomolybdenum assay, the cupric ion reducing (CUPRAC) method, the ferric reducing antioxidant power (FRAP) method, and metal chelating activity on ferrous ions were employed to evaluate the antioxidant capacity of *Z. taurica* subsp. *taurica* extracts.

Results obtained for all different extracts are reported in Table 3. As it was observed that the properties of the extracting solvents significantly affected total phenolic content and antioxidant capacity [21], comparing the antioxidant activity of the different extracts will enable us to establish a standardized procedure for sample preparation. All assays showed that methanol and aqueous extracts showed the highest antioxidant capacity. This observed antioxidant activity could be due to the greater presence of secondary bioactive metabolites belonging to the flavonoids noticed in the methanol extract.

#### 3.3.2. Enzyme Inhibitory Activities

Several papers have already shown the beneficial activity of phenolic compounds on tyrosinase and α-amylase inhibition [2,14,23].

All the extracts of *Z. taurica* subsp. *taurica* inhibited the studied enzymes (Table 4). ZTT-EtOAc extract presented the highest inhibition activity against tyrosinase (1.37 mg/mL) followed by ZTT-MeOH extract (1.46 mg/mL), with the ZTT-W extract exhibiting the lowest inhibition activity (2.29 mg/mL). Kojic acid was used as a reference. Acarbose, which is considered a strong inhibitor against α-amylase, was used as a reference in this enzyme inhibitory assay. Against α-amylase, ZTT-EtOAc extract presented the highest inhibition activity (1.82 mg/mL) followed by ZTT-MeOH extract (2.69 mg/mL). ZTT-W extract again demonstrated the lowest inhibition activity (62.56 mg/mL). Acarbose was used as a reference.

These results, in accordance with previously work [10], suggest that the phytochemicals responsible for tyrosinase and α-amylase inhibition could have low polarity (non-polar extracts showed higher activity).

### 3.4. Correlations among Phenolic Compounds and Assays

The correlation between phenolic compounds and biological assays (antioxidant and enzyme inhibitor assays) is reported in Table 5, where the Pearson correlation coefficients are shown.

Correlations between total phenolic and flavonoid compounds and tyrosinase assay were 0.97 and 0.75, respectively. Similarly, the correlation between total phenolic and flavonoid compounds, and α-amylase assay were 0.99 and 0.61, respectively. Correlations between total phenolic compounds and antioxidant capacity methods CUPRAC and phosphomolybdenum were 0.89 and 0.56, respectively. Correlations between total flavonoid content and antioxidant capacity methods CUPRAC, FRAP, DPPH, and phosphomolybdenum were 0.88, 0.33, 0.34, and 0.99, respectively. 

## 4. Conclusions

The present study examined the phenolic composition, the antioxidative activity, and the enzyme inhibitory activity against α-amylase and tyrosinase from *Z. taurica* subsp. *taurica* extracts using three different solvents. All extracts showed high phenolic content. We showed a significant antioxidant capacity and an inhibitory capacity against α-amylase and tyrosinase enzymes. *Ziziphora taurica* subsp. *taurica* can be considered a powerful natural antioxidant. To date, some of the studies concerning *Ziziphora* have been conducted *in vitro*, so in vivo investigations of this species are necessary in future studies.

## Figures and Tables

**Table 1:** Abbreviation, extraction yield, and total phenolic and flavonoid content of the solvent extracts from *Z. taurica* subsp. *taurica* ^x^.

Extracts	Abbreviation	Yield (%)	Total Phenolics (mg GAEs/g extract)	Total Flavonoids (mg QEs/g extract)
Ethyl Acetate	ZTT-EtOAc	3.23	34.82 ± 0.79 ^a^	20.87 ± 1.38 ^b^
Methanol	ZTT-MeOH	8.93	27.49 ± 0.74 ^b^	37.39 ± 2.54 ^a^
Water	ZTT-W	13.69	15.10 ± 0.45 ^c^	7.08 ± 0.58 ^c^

^x^ Within each column, means sharing the different superscripts show a comparison between the extracts by Tukey’s test at *p* < 0.05. GAEs and QEs: gallic acid and quercetin equivalents, respectively.

**Table 2:** Concentration (µg/g extract) and analytical characteristics of selected phytochemicals in the solvent extracts from *Z. taurica* subsp. *taurica* ^x^.

Compound	ZTT-EtOAc	ZTT-MeOH	ZTT-W	Linear Equation	R^2^	LOD (μg/L)	LOQ (μg/L)
(-)-Epicatechin	nd	nd	nd	y = 9.11x−9.99	0.9971	1.85	6.18
(+)-Catechin	nd	11.79 ± 0.21	nd	y = 1.45x+1.95	0.9974	3.96	13.20
2,5-Dihydroxybenzoic Acid	15.98 ± 0.84 ^c^	60.48 ± 1.77 ^b^	268.65 ± 5.24 ^a^	y = 3.79x−14.12	0.9980	2.12	7.08
2-Hydroxycinnamic Acid	nd	nd	nd	y = 16.72x−26.94	0.9996	0.61	2.03
3,4-Dihydroxyphenylacetic Acid	nd	3.20 ± 0.21 ^b^	17.87 ± 0.11 ^a^	y = 5.13x−12.39	0.9990	1.35	4.51
3-Hydroxybenzoic Acid	10.75 ± 0.38 ^a^	9.16 ± 0.58 ^ab^	8.12 ± 0.72 ^c^	y = 3.69x−12.29	0.9991	1.86	6.20
4-Hydroxybenzoic Acid	74.92 ± 0.60 ^b^	76.71 ± 0.88 ^b^	94.84 ± 1.44 ^a^	y = 7.62x+22.79	0.9996	1.72	5.72
Apigenin	1457.99 ± 24.48 ^a^	1270.90 ± 0.30 ^b^	201.25 ± 3.53 ^c^	y = 11.29x+38.05	0.9987	0.96	3.20
Apigenin 7-glucoside	293.07 ± 1.22 ^b^	847.22 ± 5.96 ^a^	nd	y = 21.33x−31.69	0.9983	0.41	1.35
Caffeic acid	17.42 ± 0.07 ^c^	44.12 ± 0.92 ^b^	415.94 ± 2.44 ^a^	y = 11.09x+16.73	0.9997	3.15	10.50
Chlorogenic Acid	48.33 ± 13.46 ^c^	136.14 ± 1.56 ^b^	2135.92 ± 2.02 ^a^	y = 12.14x+32.34	0.9995	0.55	1.82
Eriodictyol	82.66 ± 0.31 ^a^	31.32 ± 0.54 ^b^	7.39 ± 0.25 ^c^	y = 14.24x−0.50	0.9998	0.80	2.68
Ferulic Acid	1478.13 ± 24.89 ^b^	1540.91 ± 1.31 ^a^	1118.92 ± 7.20 ^c^	y = 3.32x−4.30	0.9992	1.43	4.76
Gallic Acid	nd	4.13 ± 0.10 ^a^	3.66 ± 0.07 ^b^	y = 4.82x−26.48	0.9988	1.46	4.88
Hesperidin	63.47 ± 3.16 ^c^	2389.83 ± 16.55 ^a^	158.20 ± 0.13 ^b^	y = 5.98x+0.42	0.9993	1.73	5.77
Hyperoside	117.67 ± 0.07 ^c^	956.75 ± 3.28 ^a^	139.13 ± 4.04 ^b^	y = 16.32x−1.26	0.9998	0.99	3.31
Kaempferol	nd	nd	3.17 ± 0.36	y = 0.82x−3.06	0.9959	3.30	10.99
Luteolin	5347.32 ± 54.96 ^a^	5339.91 ± 72.76 ^a^	780.53 ± 32.25 ^b^	y = 8.96x+26.80	0.9992	1.34	4.46
Luteolin 7-glucoside	270.77 ± 3.42 ^b^	1281.14 ± 39.03 ^a^	9.80 ± 0.66 ^c^	y = 45.25x+156.48	0.9996	0.45	1.51
*p*-Coumaric Acid	97.06 ± 1.72 ^c^	109.55 ± 0.73 ^b^	174.73 ± 2.53 ^a^	y = 17.51x+53.73	0.9997	1.93	6.44
Pinoresinol	262.78 ± 0.40 ^a^	79.11 ± 0.85 ^c^	107.54 ± 0.52 ^b^	y = 0.80x−2.69	0.9966	3.94	13.12
Protocatechuic Acid	74.35 ± 1.88 ^c^	175.75 ± 1.86 ^b^	378.73 ± 1.26 ^a^	y = 5.65x−9.99	0.9990	1.17	3.88
Pyrocatechol	nd	nd	nd	y = 0.11x−0.52	0.9916	9.62	32.08
Quercetin	nd	12.82 ± 0.20 ^b^	57.12 ± 0.70 ^a^	y = 14.68x−18.25	0.9997	1.23	4.10
Rosmarinic Acid	29.39 ± 1.49 ^c^	524.65 ± 14.24 ^b^	2074.40 ± 3.55 ^a^	y = 9.82x−17.98	0.9989	0.57	1.89
Sinapic Acid	449.82 ± 15.76 ^a^	35.79 ± 0.30 ^b^	23.75 ± 0.77 ^b^	y = 2.09x−6.79	0.9974	2.64	8.78
Syringic Acid	76.05 ± 3.45 ^c^	103.67 ± 2.51 ^b^	157.88 ± 2.09 ^a^	y = 0.74x−1.54	0.9975	3.75	12.50
Taxifolin	nd	nd	0.95 ± 0.32	y = 12.32x+9.98	0.9993	1.82	6.05
Vanillic Acid	694.01 ± 64.38 ^a^	735.80 ± 24.67 ^a^	476.23 ± 9.98 ^b^	y = 0.49x−1.61	0.9968	2.56	8.54
Vanillin	31.36 ± 1.39 ^a^	15.89 ± 0.29 ^b^	nd	y = 2.02x+135.49	0.9926	15.23	50.77
Verbascoside	41.47 ± 12.61 ^a^	4.57 ± 0.31 ^b^	41.99 ± 0.66 ^a^	y = 8.59x−28.05	0.9988	0.82	2.75

^x^ Within each row, means sharing the different superscripts show a comparison between the samples by Tukey’s test at *p* < 0.05. nd: not detected. LOD and LOQ: limit of detection and limit of quantification, respectively.

**Table 3:** Antioxidant activities of standards and the extracts from *Z. taurica* subsp. *taurica* ^x^.

Assays	ZTT-EtOAc	ZTT-MeOH	ZTT-W	Trolox	EDTA
*Inhibition Concentration (IC_50_: mg/mL)*					
DPPH Radical Scavenging	15.75 ± 0.66 ^c^	5.74 ± 0.08 ^b^	7.02 ± 0.23 ^b^	0.25 ± 0.01 ^a^	-
ABTS Radical Scavenging	6.30 ± 0.03 ^d^	2.74 ± 0.10 ^c^	2.39 ± 0.10 ^b^	0.26 ± 0.01 ^a^	-
Phosphomolybdenum	2.60 ± 0.03 ^c^	1.84 ± 0.08 ^b^	3.80 ± 0.17 ^d^	1.15 ± 0.01 ^a^	-
CUPRAC Reducing Power	2.39 ± 0.10 ^b^	2.24 ± 0.11 ^b^	2.80 ± 0.02 ^c^	0.31 ± 0.02 ^a^	-
FRAP Reducing Power	3.41 ± 0.28 ^c^	1.42 ± 0.04 ^b^	1.71 ± 0.02 ^b^	0.1 ± 0.01 ^a^	-
Ferrous Ion Chelating	51.40 ± 1.32 ^c^	3.77 ± 0.09 ^b^	1.04 ± 0.01 ^b^	-	0.034 ± 0.003 ^a^
*Antioxidant Activity*					
DPPH Radical Scavenging (mg TE/g extract)	15.01 ± 0.68 ^c^	42.15 ± 0.6 1^a^	33.99 ± 1.21 ^b^	-	-
ABTS Radical Scavenging (mg TE/g extract)	42.06 ± 0.22 ^c^	97.01 ± 3.45 ^b^	111.94 ± 4.74 ^a^	-	-
Phosphomolybdenum (mg TE/g extract)	446.22 ± 4.94 ^b^	630.08 ± 28.80 ^a^	304.30 ± 13.99 ^c^	-	-
CUPRAC Reducing Power (mg TE/g extract)	131.89 ± 5.95 ^a^	134.66 ± 7.00 ^a^	105.47 ± 0.70 ^b^	-	-
FRAP Reducing Power (mg TE/g extract)	30.79 ± 2.55 ^c^	73.62 ± 1.85 ^a^	61.27 ± 0.67 ^b^	-	-
Ferrous Ion Chelating (mg EDTAE/g extract)	1.03 ± 0.04 ^c^	18.53 ± 0.47 ^b^	69.31 ± 0.18 ^a^	-	-

^x^ Within each row, means sharing the different superscripts show a comparison between the samples by Tukey’s test at *p*<0.05. TE and EDTA: Trolox and Ethylenediaminetetraacetic acid (disodium salt) equivalents, respectively.

**Table 4:** Enzyme inhibition activities of standards and the solvent extracts from *Z. taurica* subsp. *taurica* ^x^.

Assays	ZTT-EtOAc	ZTT-MeOH	ZTT-W	Kojic acid	Acarbose
*Inhibition Concentration (IC_50_: mg/mL)*					
Tyrosinase Inhibition	1.37 ± 0.07 ^b^	1.46 ± 0.06 ^b^	2.29 ± 0.13 ^c^	0.37 ± 0.02 ^a^	-
α-Amylase Inhibition	1.82 ± 0.08 ^ab^	2.69 ± 0.14 ^b^	62.56 ± 0.56 ^c^	-	1.21 ± 0.07 ^a^
*Enzyme inhibition activity*					
Tyrosinase Inhibition (mg KAE/g extract)	262.76 ± 13.82 ^a^	246.27 ± 9.75 ^a^	156.88 ± 8.88 ^b^	-	-
α-Amylase Inhibition (mg ACE/g extract)	672.87 ± 28.68 ^a^	452.44 ± 23.81 ^b^	16.03 ± 0.18 ^c^	-	-

^x^ Within each row, means sharing the different subscripts show a comparison between the samples by Tukey’s test at *p* < 0.05. KAE and ACE: kojic acid and acarbose equivalent.

**Table 5:** Correlations among phenolic compounds and assays ^x^.

Assays and Compounds	Tyrosinase	α-Amylase	CUPRAC	FRAP	ABTS	DPPH	Phosphomolybdenum	Ferrous Ion Chelating
α-Amylase	0.982	1						
CUPRAC	0.973	0.912	1					
FRAP	–0.376	–0.544	–0.153	1				
ABTS	–0.766	–0.874	–0.599	0.883	1			
DPPH	–0.363	–0.533	–0.14	0.999 ^z^	0.877	1		
Phosphomolybdenum	0.736	0.595	0.872	0.350	–0.13	0.363	1	
Ferrous Ion Chelating	–0.995	–0.996	–0.944	0.470	0.829	0.458	–0.662	1
Total Flavonoid	0.751	0.612	0.883	0.330	–0.152	0.342	0.999 ^y^	–0.679
Total Phenolic	0.973	0.999 ^y^	0.895	–0.578	–0.893	–0.567	0.562	–0.992
Caffeic Acid	–0.996	–0.962	–0.989	0.295	0.709	0.282	–0.791	0.982
Vanillic Acid	0.957	0.884	0.988	–0.09	–0.543	–0.091	0.901	–0.919
Chlorogenic Acid	–0.994	–0.956	–0.992	0.273	0.693	0.260	–0.805	0.978
Sinapic Acid	0.639	0.774	0.446	–0.953	–0.984	–0.949	–0.049	–0.716
Ferulic Acid	0.960	0.890	0.999 ^y^	–0.101	–0.556	–0.088	0.896	–0.926
Luteolin 7-glucoside	0.543	0.374	0.721	0.574	0.124	0.585	0.968	–0.453
Hesperidin	0.336	0.151	0.543	0.747	0.348	0.756	0.884	–0.236
Hyperoside	0.348	0.164	0.554	0.738	0.335	0.747	0.891	–0.249
Rosmarinic Acid	–0.996	–0.995	–0.949	0.456	0.820	0.444	–0.673	0.999 ^z^
Luteolin	0.990	0.945	0.996	–0.239	–0.666	–0.226	0.826	–0.969
Apigenin	0.999 ^z^	0.981	0.975	–0.369	–0.762	–0.357	0.741	–0.994

^x^ Data show the Pearson correlation coefficients between the parameters. ^y^ Significant at *p* < 0.05; ^z^ Significant at *p* < 0.01.

## Supplementary Materials

All analytical methods applied for phenolic composition (TPC, TFC), antioxidant and enzyme inhibitory activities were given in the supplementary materials (S1). The supplementary materials are available online at https://www.mdpi.com/2218-273X/9/8/367/s1.
